# Optimism and pasture access in dairy cows

**DOI:** 10.1038/s41598-021-84371-x

**Published:** 2021-03-01

**Authors:** Andrew Crump, Kirsty Jenkins, Emily J. Bethell, Conrad P. Ferris, Helen Kabboush, Jennifer Weller, Gareth Arnott

**Affiliations:** 1 Centre for Philosophy of Natural and Social Science, London School of Economics and Political Science, London, UK; 2 Institute for Global Food Security, School of Biological Sciences, Queen’s University, Belfast, UK; 3 School of Biological and Environmental Sciences, Liverpool John Moores University, Liverpool, UK; 4 Agri-Food and Biosciences Institute, Hillsborough, UK; 5 Center for Animal Welfare Science, Purdue University, West Lafayette, USA

**Keywords:** Psychology, Zoology

## Abstract

Allowing dairy cattle to access pasture can promote natural behaviour and improve their health. However, the psychological benefits are poorly understood. We compared a cognitive indicator of emotion in cattle either with or without pasture access. In a crossover experiment, 29 Holstein–Friesian dairy cows had 18 days of overnight pasture access and 18 days of full-time indoor housing. To assess emotional wellbeing, we tested cows on a spatial judgement bias task. Subjects learnt to approach a rewarded bucket location, but not approach another, unrewarded bucket location. We then presented cows with three “probe” buckets intermediate between the trained locations. Approaching the probes reflected an expectation of reward under ambiguity—an “optimistic” judgement bias, suggesting positive emotional states. We analysed the data using linear mixed-effects models. There were no treatment differences in latency to approach the probe buckets, but cows approached the known rewarded bucket slower when they had pasture access than when they were indoors full-time. Our results indicate that, compared to cattle housed indoors, cattle with pasture access display less anticipatory behaviour towards a known reward. This reduced reward anticipation suggests that pasture is a more rewarding environment, which may induce more positive emotional states than full-time housing.

## Introduction

Picture the scene: Holstein–Friesian cows grazing lush pasture on a summer afternoon. Consumers overwhelmingly support such pasture-based systems and oppose housing cattle indoors full-time. Ninety-five percent of the British public believe pasture access benefits dairy cows^1^. That figure is 88% in Germany^2^, 84% in the United States and Canada^3^, and 81% in Brazil^4^. Industry stakeholders also value pasture access, noting its importance for cattle welfare^3,5^. However, housing cows indoors full-time facilitates feeding high-energy diets and increasing herd size on the same geographical area^6^. As a result, farmers across Europe and North America increasingly keep cattle indoors all year round^6,7^. In Denmark, Greece, and Poland, less than a quarter of dairy cows went out to pasture in 2019^7^. In the United States, only 20% of lactating cows and 34% of dry cows accessed pasture in 2013^8^. Some countries buck this trend. Over 95% of British and Irish dairy cows went out to pasture in 2019^7^, whilst Finland, Norway, and Sweden have banned full-time housing^7,9^. Worldwide, though, most milk now comes from dairy cows without any pasture access^10^.

Dairy cattle themselves also value pasture access. Given the choice between pasture and housing, cattle usually spend longer at pasture, particularly at night^11–15^. When pasture access requires walking long distances^12,16^ or pushing weighted doors^17^, cows appear to value pasture as highly as fresh food. Lying behaviour is strongly motivated and crucial for cow comfort^18^. Cows at pasture have longer lying bouts and are less restless^19,20^. Softer lying and walking surfaces also reduce the risk of injuries^21^, lameness^20,22^, and mastitis^23,24^. Cubicle barns have limited space and localised resources, so herds at pasture are less aggressive^25^ and more synchronous^19,25^. Full-time housing has some welfare benefits, such as protection from extreme weather^26^, lower risk of gastrointestinal parasites^27^, and greater control over nutrition^28^. Despite these advantages, several recent reviews have concluded that pasture access improves dairy cattle welfare^29–32^.

However, pasture’s importance for cows’ psychological wellbeing is poorly understood. Wellbeing requires a favourable balance of positive and negative emotions^33–35^. Two dimensions characterise emotion: arousal, the intensity of activation, and valence, whether the state is pleasant or unpleasant^36–40^. In humans, for instance, both elation and terror are high-arousal states, but elation is positively valenced, whereas terror is negatively valenced. Measures of cognition, physiology, and behaviour can reveal where animals fall along the two axes^39,41^. Based on these indicators, does pasture access induce positive emotions in dairy cows?

Emotions influence cognition, including attention, judgement, and memory^42–44^. For humans, positive emotions cause more optimistic judgements about ambiguous stimuli (“judgement bias”)^45–49^. Optimism also indicates psychological wellbeing in animals^50^, from primates to insects (reviews and meta-analyses:^51–55^). When presented with ambiguous stimuli, animals in positive-valence states expect more positive outcomes than animals in negative-valence states. To measure this judgement bias, researchers train subjects to respond differently to two unidimensional stimuli (e.g. spatial locations^56^). One stimulus (P) signals a relatively positive outcome, whereas the other stimulus (N) signals a relatively negative outcome. After training, subjects are exposed to ambiguous intermediate stimuli (probes). P-like responses to the probes indicate that the animal expects a positive outcome (i.e. optimism), whereas N-like responses indicate that the animal expects a negative outcome (i.e. pessimism). In a meta-analysis of 71 studies on 22 species, Lagisz et al*.*^52^ linked better housing and husbandry to more optimistic judgements about ambiguous stimuli.

Judgement biases are a popular indicator of livestock emotions and welfare^57^. For example, Neave et al*.*^58^ trained dairy calves to respond differently to red and white screens. “Go” responses (nose-touching) to one colour (P) yielded a milk reward, whilst “No-go” responses to the other colour (N) averted a one-minute time-out. When subsequently tested on ambiguous probe colours (pink screens), calves made more Go (i.e. optimistic) responses before hot-iron disbudding than after (see also^59^). In other calf studies, maternal separation induced pessimism^60^, and pair-housing induced optimism^61^. Pasture access also led to optimistic judgement biases in horses^62,63^. To our knowledge, judgement bias has not been investigated in adult cattle, but this method could reveal whether pasture access influences cows’ psychological wellbeing^29^.

The present crossover study measured emotional wellbeing in cows, which were given 18 days of overnight pasture access (PAS treatment) and 18 days of full-time housing (PEN treatment). We trained subjects on a spatial Go/No-go task, where a bucket at one location (P) contained food and a bucket at another location (N) was empty. Go responses and short response latencies to three intermediate probe locations indicated optimistic judgement biases. We hypothesised that cows in the PAS treatment would make more Go responses and have shorter response latencies to the probes than cows in the PEN treatment, indicating greater emotional wellbeing. We also predicted that likelihood to respond to the probe buckets would decrease—and latency would increase—with day number, as subjects learnt that the probes were unreinforced^65^. This was, to our knowledge, the first judgement bias study on cows^64^.

## Methods

### Ethical approval

This research was approved by Queen’s University Belfast’s Animal Research Ethics Committee, School of Biological Sciences (approval number: QUB-BS-AREC-18-05). The experiment was below the threshold of procedures detailed within the Animals (Scientific Procedures) Act 1986, and animal welfare was prioritised throughout. We confirm that all methods were carried out in accordance with relevant guidelines and regulations.

### Subjects and housing

We performed this study from May to August 2018 at the Agri-Food and Biosciences Institute (AFBI), Hillsborough, County Down, Northern Ireland (54° 5′ N; 6° 1′ W). We recruited 29 lactating Holstein–Friesian dairy cows (2.7–8.7 years old, mean: 4.3 years; 209–273 days calved, mean: 241 days). Subjects were managed alongside three non-study individuals, which maintained a 1:1 cow/cubicle ratio, giving a total herd size of 32 animals. Every cow had been at pasture that spring but was cubicle-housed for 8 weeks before the experiment began. During this time, the herd was managed as one group. Cows were kept in two interconnected pens (pen dimensions: 113.1 m^2^; 13.3 × 8.5 m), each containing 16 cantilever-type cubicles in three rows. Two of the rows, arranged “head-to-head”, had five cubicles each (cubicle dimensions: 2.2 m × 1.2 m); the third row, facing the pens’ back wall, had six cubicles (cubicle dimensions: 2.5 m × 1.2 m). Every cubicle contained a Pasture Mat rubber mat (Promat, Woodstock, Canada). The pens also had concrete standing and walking areas, which were scrape-cleaned six times daily. Subjects were given ad libitum access to fresh water and ad libitum grass silage delivered at approximately 09.00. Every day, milking occurred at 06.30 and 15.00 in a Boumatic Daytona RMS-X Exterior 50-Point Rotary Parlour.

### Timeline and treatments

We carried out judgement bias training from 24th May to 18th June 2018, judgement bias inclusion testing from 19th to 22nd June 2018, and the experiment from 23rd June to 3rd August 2018. The day before the experiment, we separated the pens and pseudorandomly divided subjects into two herds. Plywood visual barriers were installed to limit emotional contagion between treatments^66^. We balanced the groups for mobility scores and P side (left/right) in the judgement bias task. To account for mobility, a veterinary graduate (H.K.) assessed each animal’s gait 4 days before the experiment began (following^67^). Both groups had equal numbers of non-lame (scores: 0 or 1) and lame animals (scores: 2 or 3; see^19^ for details).

This experiment used a two-period crossover design with repeated measures. The first period was from 25th June to 13th July 2018 (18 days); the second period was from 16th July to 3rd August 2018 (18 days). There were two parallel treatments: the PAS treatment had daily pasture access from 16.00 to 10.00, whilst the PEN treatment lived in cubicle housing 24 h per day (except during milking). PAS cows were managed in a rotational grazing system, with grazing area 1370–3950 m^2^ (depending on herbage mass available) and distance to parlour 190–295 m. During each experimental period, we cut grass samples three times (six total). Herbage was high quality: mean oven dry matter (DM) content was 239 (SD 9) g/kg in the first period and 215 (SD 9) g/kg in the second period; mean crude protein content was 226 (SD 12) g/kg DM in the first period and 207 (SD 12) g/kg DM in the second period; and mean metabolizable energy content was 12.0 MJ/kg DM in the first period and 10.9 MJ/kg DM in the second period.

We collected judgement bias data during the daytime (10.00–15.00), when both groups were kept indoors with equivalent silage provision. The group in the PAS treatment during the first experimental period (PAS-first) contained 14 study animals (2.7–8.7 years old, mean: 4.5 years; 219–260 days calved, mean: 240 days). The group in the PEN treatment during the first experimental period (PAS-second) contained 15 study animals (2.7–7.8 years old, mean: 4.2 years; 209–273 days calved, mean 242 days). With the three non-study animals, both groups contained 16 individuals. The second experimental period was identical to the first, except the groups swapped treatments.

### Judgement bias

**Figure 1:**
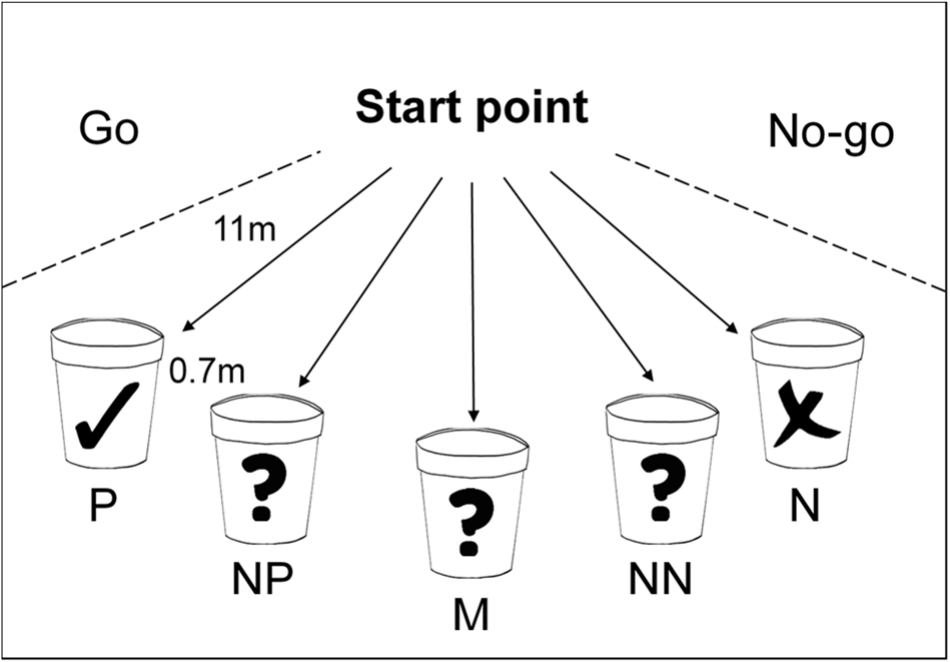
Diagram of the experimental setup, illustrating the five bucket locations (positive, P; near-positive, NP; middle, M; near-negative, NN; negative, N) and trained responses (Go, No-go).

Judgement bias testing involved two pens adjacent to the home pens: the holding area, where subjects were kept before sessions and during inter-trial intervals, and the testing area (13.3 × 3.1 m), where the task was carried out. Subjects in the holding area could not see the testing area. Once per weekday, experimenter 1 (A.C.) individually moved each cow into the holding area (subject order randomised each day). We used a spatial Go/No-go judgement bias task, with a bucket at one of five locations^56,68,69^ (Fig. 1). The P and N stimuli were buckets at the right and left locations (location counterbalanced between subjects). Rewarded P buckets contained 130 g of grain-based concentrate feed, which cattle find very desirable^70^. N buckets were not reinforced. The ambiguous probe stimuli were buckets at three intermediate spatial locations; these were also not reinforced. We ended trials if subjects did not make a Go response within 20 s. If the subject made a Go response and the bucket was rewarded, we allowed an additional 30 s to feed. We pseudorandomised trial order—subjects never had more than two consecutive buckets at the same location. Between trials, experimenter 1 moved the cow back into the holding area, and experimenter 2 (K.J.) re-set the bucket. Experimenter 2 filmed sessions on a tripod-mounted Sony HDR-CX450 1080p Camcorder.

Before the experiment began, cows underwent 18 training days. We divided these into six blocks of 3 days (Table 1), with one session of consecutive trials per subject per day. By the final 3-day training block, each cow received two rewarded P trials (P-Rew), one unrewarded P trial (P-Unr), and three unrewarded N trials (N-Unr) per day across a six-trial session. P-Unr trials introduced a one-third variable reinforcement ratio. Variable reinforcement reduces extinction learning towards unrewarded probes, which can look like increased pessimism without any change in emotional state^65^. To maintain task motivation, subjects never received more than two consecutive unreinforced trials (either P-Unr or N-Unr), and the last P trial was always P-Rew.

**Table 1:** Training timeline, with the number of rewarded P trials (P-Rew), unrewarded P trials (P-Unr), and unrewarded N trials (N-Unr) per cow in each consecutive three-day block.

Days	P-Rew trials	P-Unr trials	N-Unr trials	Total trials
1–3	1	0	0	1
4–6	2	0	0	2
7–9	2	0	1	3
10–12	2	0	2	4
13–15	2	0	3	5
16–18	2	1	3	6

After the training phase, we carried out 3 days of inclusion testing to confirm that subjects had learnt the spatial discrimination task. We recorded responses in six inclusion trials per day (18 trials total). Three trials per day involved the P location (2 × P-Rew; 1 × P-Unr) and three trials per day involved the N location (3 × N-Unr). For each subject, we extracted the latency for all 18 trials, with No-go responses given a ceiling latency of 20 s. Our inclusion criteria are outlined in the “Statistical analyses” section.

During both experimental phases, we carried out judgement bias testing every Monday, Wednesday, and Friday (8 × testing sessions per individual per phase; 16 × testing sessions per individual total). Half of testing sessions included three P trials (2 × Rew; 1 × Unr) and two N trials, whilst the other half included two P trials (2 × Rew) and three N trials. The remaining trial was a probe bucket at one of three equidistant intermediate locations: near-positive (NP; 0.7 m from P), middle (M; 1.4 m from both P and N), and near-negative (NN; 0.7 m from N). The probe trial randomly replaced either P-Unr or N trials. Experimenter 3 (H.K.) extracted data for the P, N, and probe buckets from video footage. If the subject’s muzzle touched or entered the bucket, a Go response was recorded. Otherwise, a No-go response was recorded. Latency was also measured, from one hoof crossing a standardised start line to the Go response (distance: 11 m). Experimenter 3 was blind to both treatment (PAS/PEN) and which bucket location was rewarded (P/N). Throughout the experiment, we continued training sessions on Tuesdays and Thursdays. This increased the P/N:probe ratio, further reducing extinction learning towards the probes^51^.

### Statistical analysis

We analysed the data in R (R Core Team, Cran-r-project, Vienna, Austria, version 3.6.2). We checked data and model assumptions using histograms and qqplots, applying transformations where appropriate. We used the package “lme4”^71^ to run mixed-effects models and dropped interactions when this reduced the model’s Akaike Information Criterion value by > 5. We then extracted *p* values using type III Wald’s tests. Where factors had multiple levels or interactions involved multiple comparisons, we performed a Tukey’s post-hoc test (“lsmeans” package^72^) to quantify differences between levels or comparisons. Unless otherwise stated, we present data as means ± standard error of the mean.

For the training data, we used a statistical inclusion criterion. We ran a Wilcoxon test on the latency data from each cow’s inclusion trials (*n* = 18; 9 × P, 9 × N). To proceed, subjects needed shorter response latencies to the P location than the N location (*p* < 0.05). We also ran a general linear mixed-effects model on the inclusion data to establish that subjects learnt the left/right association, rather than using olfactory cues to approach the rewarded location. Latency was included in the model as the response variable; location/reward category (P-Rew, P-Unr, N-Unr) was included as a fixed effect; and cow ID was included as a random effect. We identified differences between categories with a Tukey’s post-hoc test. Subjects not using olfactory cues would show no difference between P-Rew and P-Unr, but a difference between P and N; subjects using olfactory cues would show no difference between P-Unr and N-Unr, but a difference between Rew and Unr.

For the judgement bias data, we ran models with both binary Go/No-go responses and response latency as the dependent variables^52^. We fitted a generalised linear mixed-effects model for the Go/No-go data (binomial distribution, logit link). We ran a general linear mixed-effects model for the latency data, which we transformed by taking the natural logarithm of the value + 1. We excluded No-go responses from this model. In both models, the fixed effects were housing treatment (PAS, PEN), treatment order (PAS-first, PAS-second), bucket location (P, NP, M, NN, N), and day number (1–16). We also included treatment × treatment order, treatment × bucket location, and bucket location × day number interactions as fixed effects. Cow ID was included as a random effect. We also ran a separate model on latency (log-transformed + 1) to the P location only. Fixed and random effects were the same as for the previous model, except that we removed bucket location. To account for food motivation, we also included body condition score and time of day as fixed effects, as well as body condition score × treatment and time of day × treatment interactions.

## Results

### Judgement bias training

During inclusion testing, all 29 cows approached the P location faster than the N location (all subjects: *p* < 0.001) and advanced to the experimental phase. Investigating the effect of bucket location and food reward presence/absence, we found a difference in latency between P-Rew trials (median latency ± SD: 5.75 ± 0.92 s), P-Unr trials (5.75 ± 0.93 s), and N-Unr trials (20 ± 4.36 s; *χ*^*2*^_2_ = 2248, *p* < 0.001). Post-hoc tests revealed no difference between P-Rew and P-Unr (*z* =  − 0.14, *p* = 0.989). However, subjects were faster to both P-Rew (*z* =  − 42.34, *p* < 0.001) and P-Unr (*z* =  − 33.62, *p* < 0.001), compared to N-Unr.

### Judgement bias testing

We collected data from 2741 judgement bias trials. Excluding the 1342 No-go trials, latency data were available from 1399 Go trials. Latency from start line to bucket ranged from 2.75 to 18.91 s (mean: 7.03 s).

**Figure 2:**
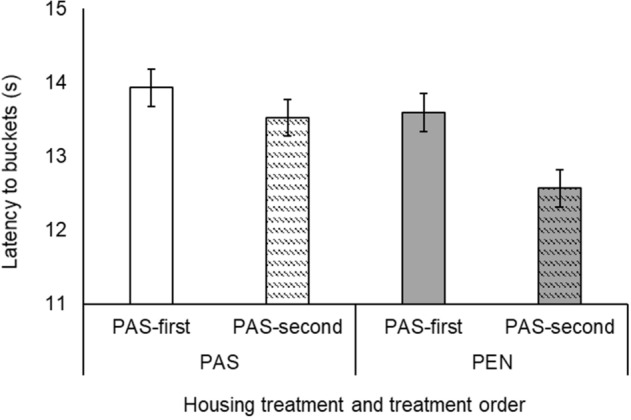
Interaction between housing treatment (pasture access: PAS; cubicle housing: PEN) and treatment order (PAS-first, PAS-second) in response latency to all five bucket locations. Error bars represent standard error of the mean.

Cows in the PAS treatment were less likely to approach the buckets (PAS: 47.75% trials; PEN: 53.24% trials; *χ*^2^_1_ = 9.90, *p* < 0.001) and took longer to do so (PAS: 7.12 ± 0.07 s; PEN: 6.42 ± 0.05 s; *χ*^2^_1_ = 26.91, *p* < 0.001). Treatment order did not affect approach likelihood (*χ*^2^_1_ = 2.35, *p* = 0.13) or latency (*χ*^2^_1_ = 0.38, *p* = 0.54). There was a treatment × treatment order interaction for both likelihood to approach (*χ*^2^_1_ = 14.99, *p* < 0.001) and latency to approach (*χ*^2^_1_ = 6.08, *p* = 0.01; Fig. 2). During the PEN treatment, the PAS-first group had a smaller increase in approach likelihood and reduction in approach latency than the PAS-second group.

**Figure 3:**
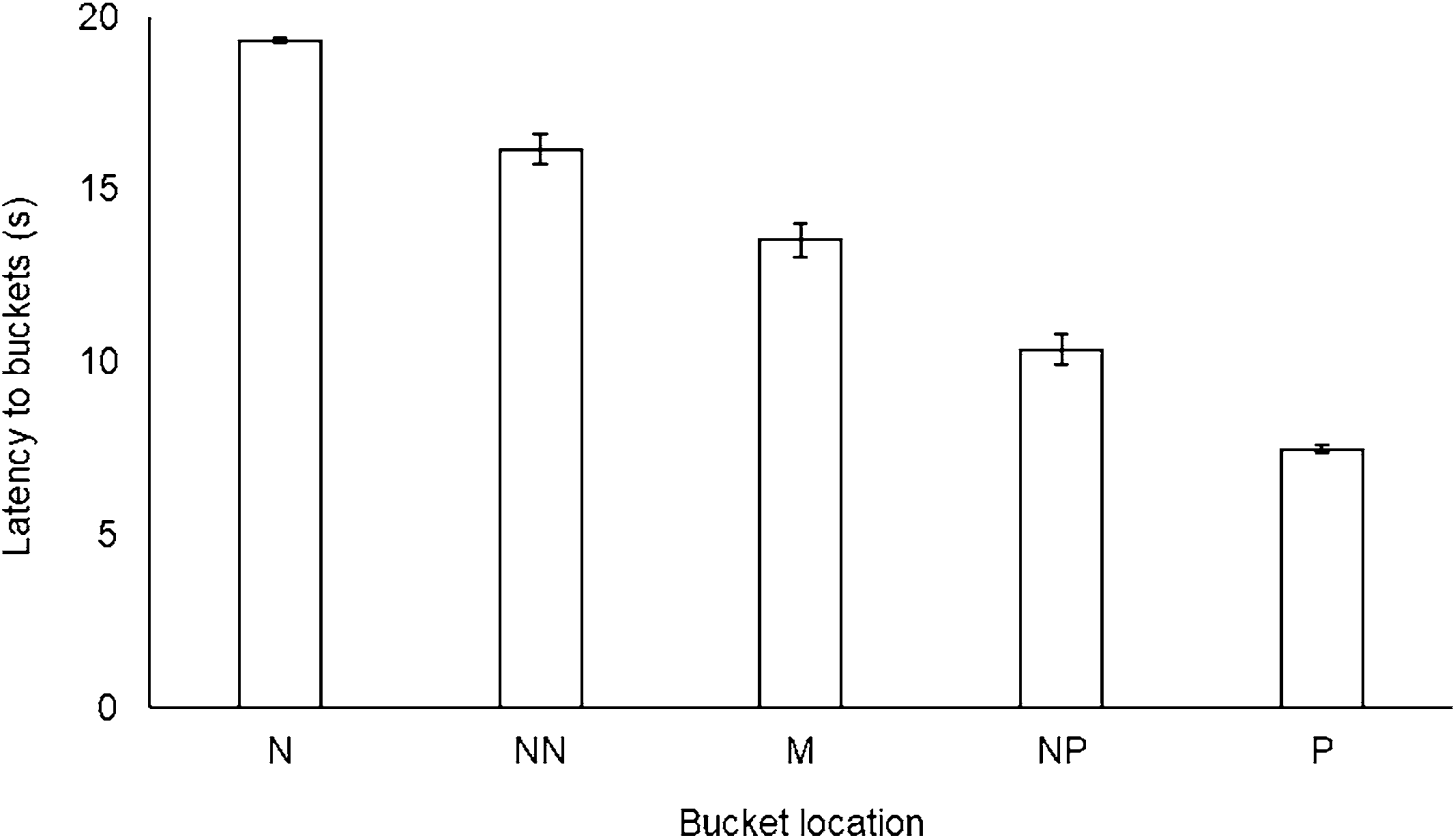
Response latency to the five bucket locations throughout the experiment (negative: N; near-negative: NN; middle: M; near-positive: NP; positive: P). Error bars represent standard error of the mean.

**Figure 4:**
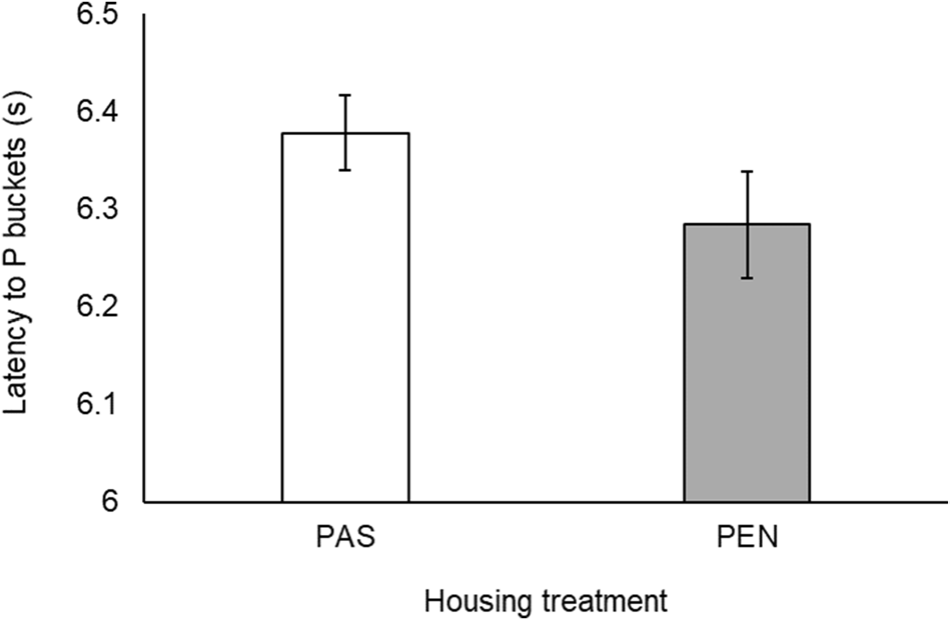
Response latency to the positive (P) bucket location in each housing treatment (pasture access: PAS; cubicle housing: PEN). Error bars represent standard error of the mean.

There was an effect of bucket location on both the number of Go responses (*χ*^2^_4_ = 816.31, *p* < 0.001) and approach latency (*χ*^2^_4_ = 1089.89, *p* < 0.001; Fig. 3). Post-hoc tests revealed that all five bucket locations differed from one another in terms of both approach likelihood and latency (Table 2). There was no treatment × bucket location interaction for approach likelihood (*χ*^2^_4_ = 2.11, *p* = 0.72), but there was an interaction for approach latency (*χ*^2^_4_ = 15.87, *p* < 0.005). This showed that the main effect of treatment on latency was localised to the P location: cows were slower to approach P when they were in the PAS treatment than the PEN treatment (PAS: 6.38 ± 0.04 s; PEN: 6.28 ± 0.05 s; *t*_1,386_ = 6.39, *p* < 0.001; Fig. 4). There was no treatment difference in latency to any other location (NP: *t*_1,385_ = 0.42, *p* > 0.99; M: *t*_1,386_ =  − 0.66, *p* > 0.99; NN: *t*_1,387_ =  − 1.60, *p* = 0.85; N: *t*_1,387_ = 0.45, *p* > 0.99).

**Table 2:** Pairwise comparisons of the likelihood and latency to approach each bucket location, and for the bucket location × day number interaction.

Comparison	Bucket location approach				Bucket location × day number			
	Likelihood		Latency		Likelihood		Latency	
	*z*	*p*	*t*	*p*	*z*	*p*	*t*	*p*
P – NP	− 5.98	** < 0.001**	9.40	** < 0.001**	− 2.86	**0.03**	3.88	** < 0.005**
P – M	− 11.00	** < 0.001**	13.14	** < 0.001**	− 4.11	** < 0.001**	2.91	**0.03**
P – NN	− 15.52	** < 0.001**	14.70	** < 0.001**	− 2.59	0.07	− 0.22	< 1.00
P – N	− 28.23	** < 0.001**	26.29	** < 0.001**	− 2.39	0.12	− 1.34	0.67
NP – M	− 3.84	** < 0.005**	4.48	** < 0.001**	− 0.93	0.88	− 0.20	< 1.00
NP – NN	− 7.73	** < 0.001**	7.26	** < 0.001**	0.42	0.99	− 2.46	0.10
NP – N	− 16.78	** < 0.001**	15.52	** < 0.001**	1.21	0.75	− 3.81	** < 0.005**
M – NN	− 4.17	** < 0.001**	3.09	**0.02**	1.42	0.61	− 2.11	0.22
M – N	− 13.80	** < 0.001**	10.06	** < 0.001**	2.42	0.11	− 3.20	**0.01**
NN – N	− 9.53	** < 0.001**	6.01	** < 0.001**	0.79	0.93	− 0.64	0.97

Bold *p* values are significant.

**Figure 5:**
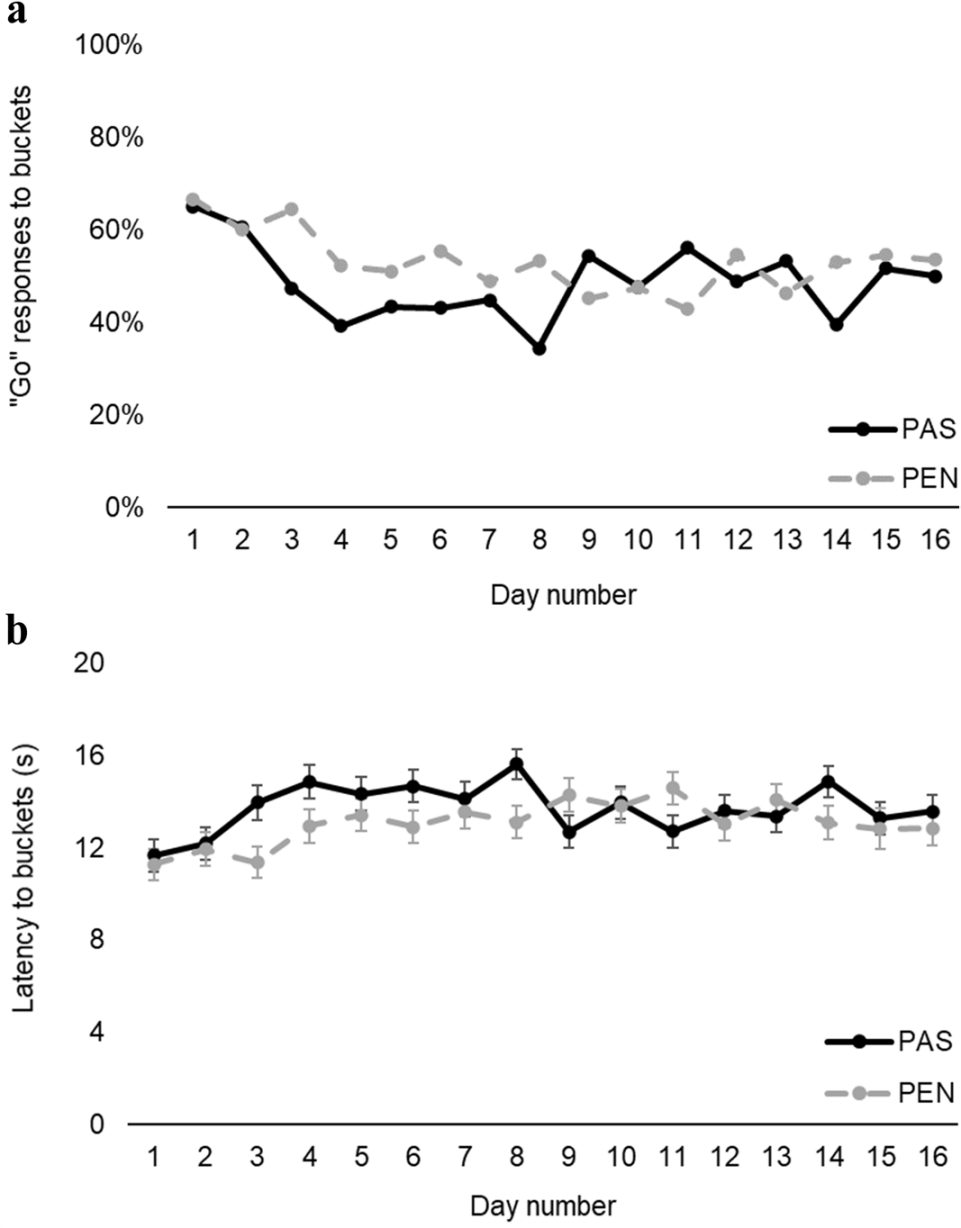
(**a**) Percentage of “Go” responses and (**b**) response latency to all buckets in each treatment (pasture access: PAS; cubicle housing: PEN) throughout the experiment (days 1–16). Error bars represent standard error of the mean.

As the study progressed (i.e. day number increased), likelihood to approach the buckets decreased (*χ*^2^_1_ = 27.62, *p* < 0.001; Fig. 5a) and approach latency increased (*χ*^2^_1_ = 19.28, *p* < 0.001; Fig. 5b). There was also a bucket location × day number interaction for approach likelihood (*χ*^2^_4_ = 21.72, *p* < 0.001) and latency (*χ*^2^_4_ = 25.83, *p* < 0.001; Table 2).

When we modelled latency to the P location, there was no effect of either time of day (*χ*^2^_1_ = 0.66, *p* = 0.42) or body condition score (*χ*^2^_1_ = 0.00, *p* = 0.96). There was no treatment × time of day (*χ*^2^_1_ = 0.53, *p* = 0.47) or treatment × body condition score interactions (*χ*^2^_1_ = 0.20, *p* = 0.66).

## Discussion

We investigated whether pasture access impacts judgement bias in dairy cows, to assess whether pasture improves emotional wellbeing. Cattle learnt a spatial judgement bias task, and optimistic responses increased with proximity to the rewarded location. However, there was no treatment difference in judgement bias. Subjects with pasture access were neither likelier nor faster to approach buckets when the reward contingency was ambiguous. Cows with pasture access nonetheless approached known rewarded buckets slower than cows kept indoors full-time.

We did not predict that cows would be slower to the P location in the PAS treatment than the PEN treatment. A core assumption of judgement bias tasks is that emotions bias decision-making when outcomes are uncertain^39,73,74^. As a result, treatment effects in judgement bias are expected towards the probes—not the trained P and N stimuli^39,73^. Most studies meet this assumption^52^ (for exceptions, see^50,75,76^). Moreover, we expected pasture access to reduce approach latency, representing a higher expectation of reward and an optimistic judgement bias. One explanation for our surprising result is that cows could have been less food motivated in the PAS treatment than the PEN treatment (e.g. see^77–79^). At pasture, cattle begin grazing at dawn (around 06.00)^80^, whereas subjects in our PEN treatment were only offered fresh silage at 09.00. PAS cows may, therefore, have fed for longer than PEN cows before testing. However, if food motivation were responsible, the effect would be strongest earliest in the day and decrease as all subjects spent longer with equivalent rations. Time of day did not affect latency to the P location. Additionally, we scored every cows’ body condition during both experimental phases. Higher scores reflect better nutrition^67^, so body condition score is inversely correlated with food motivation. We found no relationship between P latency and body condition score, suggesting that food motivation did not influence P latency. These converging lines of evidence indicate that treatment differences in nutrition were not responsible.

**Figure 6:**
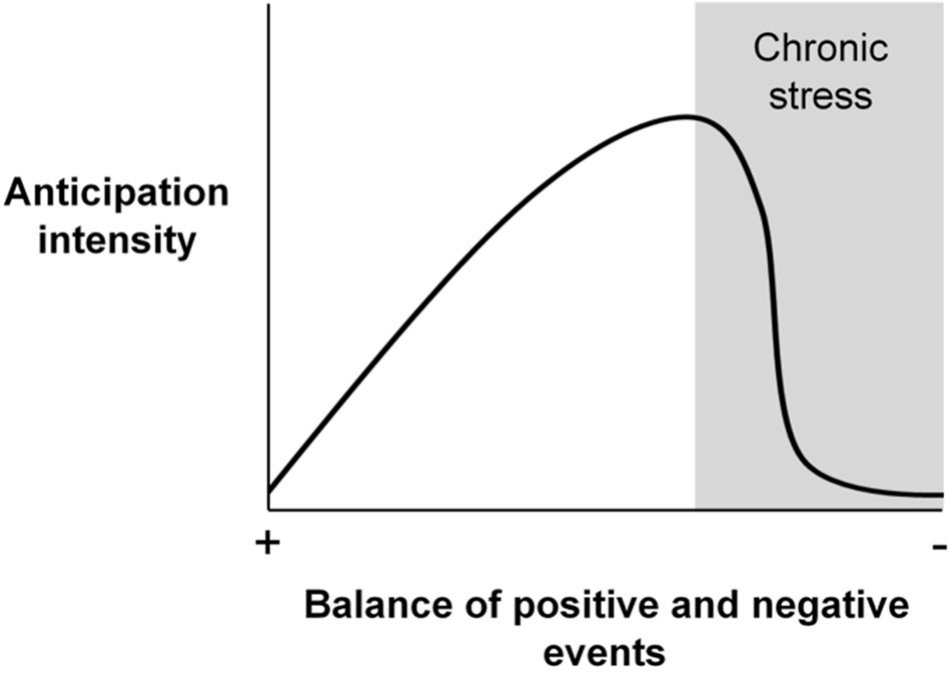
Relationship between the balance of positive and negative events in an animal’s life and anticipation intensity towards individual rewards (adapted from^96^).

We propose that reduced reward anticipation, linked to positive emotional states, explains why the PAS treatment were slower to the P location than the PEN treatment. Spruijt et al^35^ hypothesised that animals exposed to fewer, lower-quality rewards value each reward more (Fig. 6; reviewed by^81,82^; see^83^ for a critique). As an example, dairy calves in basic housing responded to a reward-predicting cue with more behavioural transitions and shorter response latencies than calves in enriched housing^84^ (see also^85^). This effect means that, in a judgement bias task, we predict opposite welfare-based differences in response patterns towards the P stimulus and the probes. If animals have received more, higher-quality rewards, the P stimulus will elicit less anticipatory behaviour, whereas the probes will elicit more^82^. Latency to a rewarded bucket meets Spruijt et al.’s definition of anticipatory behaviour: “responses elicited by rewarding stimuli that lead to and facilitate consummatory behavior” (p. 160). We, therefore, suggest that PAS cows’ longer P latencies reflected lower reward anticipation, indicating that they had more rewarding lives and better welfare, rather than pessimistic judgement biases, which would indicate less rewarding lives and worse welfare. Our earlier findings support this interpretation: compared to cows in the PEN treatment, cows in the PAS treatment had longer total lying times; fewer, longer lying bouts; and more synchronous lying behaviour^19^. These results indicate that cows at pasture were more comfortable and less restless. The inverse relationship between reward frequency and reward anticipation does not apply to chronically stressed animals, which display reduced reward valuation (anhedonia^86^). Our results indicate that PEN cows were not anhedonic but had less rewarding lives than PAS cows.

Our reward anticipation findings highlight how judgement bias tasks may quantify effects besides judgement bias^51,53^. However, few studies have compared judgement bias and reward anticipation. Optimistic judgement biases were linked to reduced anticipatory behaviour in dolphins^87^, whereas enriched housing did not affect chickens’ responses to either a judgement bias or reward anticipation task^88^. Other studies suggest treatment differences in anticipatory behaviour influenced responses to a judgement bias task. For example, disrupting reward-related behaviours in chicks reduced latencies towards the P stimulus^76^. The antidepressant reboxetine likewise reduced P responses in rats^75^, and deep-litter enrichment reduced P responses in quail^89^ (exp. 4), although neither finding was consistent across experiments. Like our results, these P response patterns can be attributed to increased reward anticipation among subjects in more negative emotional states. Moreover, because judgement bias and reward anticipation predict opposite responses, it is possible that they cancel each other out. In a meta-analysis of judgement bias studies using pharmacological manipulations, effect sizes were smaller for the P stimulus than either the probes or N stimulus^54^. To differentiate the effects of judgement bias and reward anticipation, we suggest that judgement biases are more plausible when treatment differences only occur towards the probes, whereas treatment differences localised around the P stimulus imply reward anticipation.

Despite P responses indicating that the PAS treatment induced more positive emotional states, pasture access did not influence judgement bias. This is surprising, as aversive events lead to pessimism in dairy calves^58–61^, and pasture access leads to optimism in horses^62,63^. However, studies on pigs^90^, chickens^88^, and quail^89^ have found no difference in judgement bias between housing conditions. There are two possible reasons for our null results. First, pasture access may not influence emotional state in dairy cows. This explanation might seem implausible, given pasture’s potential behavioural and health benefits, and cows’ preference and motivation for pasture^29,30^. Subjects’ behaviour during our experiment—with fewer, longer lying bouts during the PAS treatment—also indicate that PAS cows were more comfortable^19^. However, we tested judgement bias during the daytime, when both treatments were kept indoors. Pasture may only improve emotional wellbeing whilst cows are at pasture, without persisting after they go inside. Ruet et al^91^ found that, when confined indoors again, horses given pasture access rapidly returned to previous poor welfare states. Conversely, Anderson and Adolphs^92^ identified persistence as a defining feature of emotions. Their characterisation is consistent with our reward anticipation findings, which indicate that positive emotional states from overnight pasture access carried over into daytime indoor housing.

The second potential explanation for our null judgement bias results is that treatment differences in emotional state existed, but our task did not detect them. In their meta-analysis, Lagisz et al*.*^52^ identified four methodological factors that may be responsible for our findings. (1) Sex: males exhibit larger effects than females, and our population was female. (2) Stimuli: sound and tactile stimuli lead to larger effects than spatial stimuli, which we used. (3) Responses: Go/Go tasks (where both P and N require active responses) produce larger effects than Go/No-go tasks; we tested the latter. (4) Reinforcement: methods with rewarded/punished stimuli or differentially rewarded stimuli generate larger effects than rewarded/unreinforced stimuli, which we used. In addition, cognitive tasks can be inherently rewarding^93–95^. Thus, performing the judgement bias task may have itself influenced cows’ emotional state, especially in the unstimulating PEN treatment. Another potential confound in spatial judgement bias tasks is olfactory cues, which can signal reward presence. However, our cows were no faster to P-Rew buckets than P-Unr buckets, suggesting that olfaction did not play a role. Additionally, repeated judgement bias testing can increase response latencies as subjects learn the probes are unreinforced^65^. We observed this effect, but our statistical models accounted for repeated measures. Some combination of these methodological factors potentially overrode treatment differences in judgement bias.

In conclusion, giving dairy cattle pasture access appears to induce more positive emotional states than cubicle housing. We previously showed that cows are more comfortable at pasture: they exhibit longer lying times, less restlessness, and greater herd synchrony. These behaviour data are partially consistent with the present findings, collected during the same experiment. We found no difference in judgement bias between cows with and without pasture access. In our judgement bias task, however, the pasture treatment was slower to approach a known reward. This effect implies reduced reward anticipation, suggesting that cows in the pasture-based system had more rewarding lives. Collectively, our results indicate that pasture access improves emotional wellbeing in dairy cows.

## Data Availability

The datasets generated and analysed in this study are available from the corresponding author on reasonable request.
